# Rhinosectan^®^ spray (containing xyloglucan) on the ciliary function of the nasal respiratory epithelium; results of an in vitro study

**DOI:** 10.1186/s13223-018-0268-3

**Published:** 2018-10-08

**Authors:** Núria Piqué, Barbara De Servi

**Affiliations:** 1 Department of Microbiology and Parasitology, Pharmacy Faculty, Universitat de Barcelona (UB), Diagonal Sud, Facultat de Farmàcia, Edifici A, Av Joan XXIII, 27-31, 08028 Barcelona, Spain; 2 Institut de Recerca en Nutrició i Seguretat Alimentària de la UB (INSA-UB), Universitat de Barcelona, Barcelona, Spain; 3 VitroScreen Srl, Via Mosè Bianchi 103, 20149 Milan, Italy

**Keywords:** Xyloglucan, Rhinitis, Nasal obstruction, Rhinosinusitis, Barrier properties, Ciliary function, Mucociliary clearance, Mucin secretion, Allergy, Preventive measures, Medical devices

## Abstract

**Background:**

To assess the effects of Rhinosectan^®^ spray, a medical device containing xyloglucan, on nasal ciliary function (in MucilAir™Nasal cells).

**Methods:**

MucilAir™Nasal, a three-dimensional organotypic airway tissue model (with different cell types), was treated with Rhinosectan^®^ (30 µl) or with a control (saline solution). The effects of Rhinosectan^®^ were evaluated at 15 and 60 min post-exposure by: measurement of the cilia beating frequency (Hz), mucin detection (Enzyme-Linked Lectin Assay—ELLA), mucociliary clearance (µm/s) and phagocytosis assay (fluorescence).

**Results:**

Exposure of MucilAir™ to Rhinosectan^®^ did not alter the cilia beating frequency at 15 and 60 min post-exposure (diluted and undiluted). Exposure to Rhinosectan^®^ (undiluted) during 60 min increased mucociliary clearance (93.3 ± 2.1 µm/s vs. 80.9 ± 1.8 µm/s; p < 0.01) and phagocytic activity (1.89-fold increase) in comparison with saline solution. Moreover, a significant decrease in mucin concentration was observed after 15 min of exposure (171.4 ng/ml vs. 306.5 ng/ml; p < 0.01) and at 60 min post-treatment (242.7 ng/ml vs. 339 ng/ml; p < 0.05).

**Conclusions:**

The application of Rhinosectan^®^ to nasal epithelial cells does not impair ciliary movement, enhances mucociliary clearance and facilitates phagocytosis while reducing mucin secretion, which are optimal properties for the management of rhinitis and associated conditions.

## Background

Nowadays, there is growing interest in understanding the role played by the disruption of the mucosal epithelial barrier in the pathogenesis of a wide variety of diseases. Exposure to bacterial pathogens and their products, allergens, pro-inflammatory compounds or environmental particles have been identified to have role, together with the emerging evidence supporting the relevant role of microbiota changes (known as dysbiosis), promoting inflammatory conditions and leading to the development of diseases [1–4]. In this regard, there is a need to understand the specific causes of mucosal disruption of each mucosal membrane and to prevent and/or counteract their damaging effects on the mucosal membranes [1, 5].

In the case of respiratory diseases, understanding the regulation of airway epithelial barrier function is becoming a new frontier in respiratory allergic diseases, such as asthma and rhinitis, and also in respiratory infections (such as viral infections), thus opening up potential new therapeutic targets for respiratory diseases [1, 5, 6]. This is also in agreement with current recommendations of ARIA (allergic rhinitis and its impact on asthma) guidelines to avoid when possible the contact with allergens and other triggering factors as an integral part of the management strategy for rhinitis [5, 7, 8]. This could also be extended to other respiratory allergic and infectious diseases, based on current knowledge of the mechanisms by which pathogens disrupt epithelial structure and function, allowing better understanding of the associations between respiratory infections, airway inflammation and allergen sensitization [6].

In the particular case of allergic rhinitis, findings from basic science and epidemiological studies show that this disease is a part of a systemic inflammatory process and is associated with other inflammatory disorders of mucous membranes [9], including asthma, rhinosinusitis and allergic conjunctivitis [9]. In order to prevent or decrease this inflammatory process, non-pharmacological approaches that restore the barrier integrity are becoming of great interest [5, 8].

In this regard, Rhinosectan^®^ spray (Noventure, SL, Barcelona, Spain), a medical device containing xyloglucan as the main ingredient, was developed to restore the physiological functions of the nasal epithelial mucosa by forming a film that protects the mucosa from different pathogens and allergens. Rhinosectan^®^ is specifically formulated for the control and reduction of symptoms related to rhinorrea and sinus congestion due to different etiologies, such as rhinitis (seasonal, perennial allergic, infectious or vasomotor rhinitis), sinus congestion due to cold or flu; and as symptomatic relief associated to the treatment of nasal polyps and acute sinusitis. Rhinosectan^®^ spray has been shown to significantly reduce rhinorrhea, itching, TNSS (total nasal symptom score) and the severity of rhinosinusitis, in comparison with physiological saline spray, in patients with rhinosinusitis, after nasal administration during 2 weeks [10].

Xyloglucan is a natural hemicellulose extracted from the seeds of the tamarind tree (*Tamarindus indica*), which forms a bio-protective film, avoiding the contact with pathogenic bacteria, bacterial products, allergens or pro-inflammatory compounds as shown in in vitro and in vivo studies [1, 5, 11–15]. In a recent in vitro study on a MucilAir™ airway tissue model, Rhinosectan^®^ spray protected cell tight junctions (producing increases in TEER—trans-epithelial electrical resistance) and preserved the paracellular flux, even after exposure to pro-inflammatory compounds (TNF-α and LPS from *Pseudomonas aeruginosa* 10). Results obtained by confocal immunofluorescence microscopy also highlighted the barrier properties of Rhinosectan^®^, with low affectation of the tight junction proteins zonula occludens-1 and occludin, in comparison with the glucocorticoid budesonide [5].

As a continuation of this study, and taking into account the potential adverse effects of first-line drugs for the treatment of rhinitis and related diseases, such as corticosteroids and antihistamines, on the nasal structure and ciliary function [16], this study has assessed the effects of Rhinosectan^®^ on nasal functioning in a MucilAir™ airway tissue model (in terms of cilia beating frequency, mucin secretion, mucociliary clearance and phagocytic activity).

## Methods

### Cells and reagents

MucilAir™Nasal is a morphologically and functionally differentiated three-dimensional model of nasal epithelial tissue (Epithelix Sàrl, Geneva, Switzerland) [17], where the epithelia are cultivated on microporous filters at the air–liquid interface (ALI). Typical ultra-structures of the human airway epithelium are observed: tight junctions, cilia, basal cells and mucous cells. The ALI allows direct administration of an aerosol onto the apical surface, a situation that mimics the in vivo respiratory system’s exposure to aerosols [17]. Moreover, the epithelium is fed by a culture medium from the basolateral surface [17, 18]. Each batch is checked for tissue integrity (TEER measurement), as reported in the certificate of analysis. The product can be maintained in a homeostatic state for more than 1 year.

In this study, batch MP0007 was obtained from a pool of 14 healthy donors. Immediately after arrival at the laboratory, the MucilAir™ tissues were rapidly transferred to a 24-well plate previously filled with 0.7 ml of the specific MucilAir™ maintenance medium (Epithelix) at room temperature. The plates were placed overnight in an incubator at 37 °C, 5% CO_2_ and saturated humidity.

### Products

Rhinosectan^®^ spray contains physiological saline solution, methylsulfonylmethane and xyloglucan, extracted from the seeds of the tamarind tree (*Tamarindus indica*). Rhinosectan^**®**^ was kindly provided by Noventure, SL (Spain). The product has been tested pure and diluted 1:10 for all the assays performed except for the phagocytosis assay where it was only tested undiluted.

**Fig. 1:**
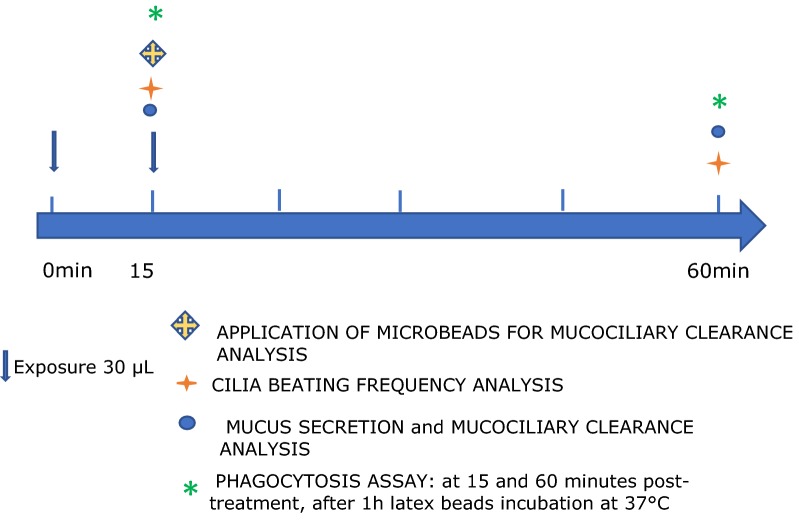
Study flow chart. The application of undiluted or tenfold diluted Rhinosectan^**®**^ is indicated with an arrow. Special characters show time points of outcome measurements at 15 and 60 min, Red Star: Cilia beating frequency; Blue Circle: Mucus secretion analysis and MCC; Green Asterisk: Phagocytic activity assay

To mimic airborne delivery, 30 μl of Rhinosectan^**®**^ was applied topically onto the surface of the epithelia (pipetted onto the surface) and the effect was tested at two time points, at 15 and 60 min post-application (Fig. 1).

As negative control, saline solution (0.9% NaCl) (Eurospital, Trieste, Italy) was used.

### Measurement of the Cilia Beating Frequency

Cilia beating frequency (CBF) and ciliated cell density were used to evaluate the normal functioning of the tissue culture. The MucilAir™ cilia beating frequency was monitored at two time points (15 and 60 min) after the addition of Rhinosectan^®^ and a saline solution as a negative control.

Morphological evaluation of the apical tissue surface was performed using an inverted microscope (Leica DMIRE2, Leica Microsystems, Germany) equipped with a digital camera. In order to record the morphology of the entire epithelium, photographs were taken. Cilia beating frequency was measured using a system that was specially designed for this purpose. The system consists of three parts: a camera (Sony XCD-V60/Firewire, Sony Cameras, USA), a PCI card and a specific software package (Cilia Beating Frequency Analyser). The software calculates the measurable areas of the images and data shown are coming from images with an active area (defined as a surface with cilia beat frequency > 3 Hz) of more than 90%. Data of cilia beating frequency represent the average value of thousands of active ciliated cells from three independent tissues, expressed as Hz.

### Mucin detection

In order to measure the amount of mucin secreted on the surface of MucilAir™, cell monolayers were treated with 30 µl of Rhinosectan^**®**^ spray or the negative control saline solution, in triplicates in a 24 well plate containing 0.7 ml of saline solution/well. After 15 and 60 min of treatment, to collect the mucus after the product treatment, 200 μl of NaCl-HEPES solution (buffered saline lavage) were applied on the apical surface of the tissue and the buffer containing mucus was collected immediately and analysed. At the different time points, the concentration of secreted mucins was measured with the Enzyme-Linked Lectin Assay (ELLA), similar to classical ELISA for protein analysis. The assay relies on the interaction of lectins with mucin glycoproteins. It allows quantitative measurement of the secreted mucins. Samples were applied to Nunc Maxisorp™ plates coated with Triticum vulgaris lectin and incubated for 30 min at 37 °C. Plates were blocked with bovine serum albumin (0.1%, w/v, in PBS) for 30 min then incubated for 30 min with horseradish peroxidase (HRP) labelled soybean lectin (1 µg/ml in PBS). HRP detection was performed using BD OptEIA™ tetramethylbenzidine (TMB) substrate, incubated at room temperature for 15 min and stopped with 2 N H_2_SO_4_. Mucin content was determined by optical absorbance (450 nm) measurement of stopped reactions.

### Mucociliary clearance analysis

The effect Rhinosectan^®^ has on mucociliary clearance (MCC) was evaluated on the MucilAir™ epithelium and measurements of the movement of microbeads were performed in triplicate, as follows. First of all, as a control, mucociliary clearance was quantified following the movement of plugs of mucus on the apical surface. Then, 30 μm polystyrene microbeads (Sigma-Aldrich Chemie GmbH, Buchs, Switzerland) contained in 30 μl of Rhinosectan^®^ or saline solution were applied to the apical surface of MucilAir™. The MCC was monitored using a high-speed acquisition camera (Sony XCD-SX910) connected to an Axiovert 200M microscope (Zeiss, Gottingen, BRD). Subsequently, 30 s movies (3 movies/insert) showing the movement of the small beads were taken and analyzed using Image Pro Plus 6.0 (Mediacy, Media Cybernetics, Rockville, Md., USA) imaging software. The first movies were acquired before the addition of the particles (control clearance) and then 15 and 60 min after the addition of Rhinosectan^®^ and the saline solution. The movement of the beads was tracked and the velocity of each particle was calculated in order to determine the mean speed of the particles as a measurement of mucociliary clearance (μm/s).

### Qualitative and quantitative phagocytosis assay

The Cayman Phagocytosis Assay Kit (IgG FITC) (Cayman Chemical Company, USA) employs latex beads coated with fluorescently-labelled rabbit-IgG as a probe for the identification of factors regulating the phagocytic process in vitro.

The assay was performed following the manufacturer’s instructions. Briefly, 100 μl of Latex Beads-Rabbit IgG-FITC solution (diluted 1:10 in saline solution) was added simultaneously with 30 μl of Rhinosectan^®^ or saline solution to MucilAir™ cell monolayers. After incubation for 15 or 60 min at 37 °C, the supernatant was carefully aspirated and cells were gently washed with assay buffer to remove unbound beads. For the qualitative analysis, cells were analyzed directly with the inverted fluorescence microscope (Leica DM IL LED FLUO, Germany) with a10× objective, which was equipped with a filter capable of measuring excitation and emission at 485 and 535 nm, respectively. For the quantitative analysis, the fluorescence intensity of the cells was measured in a fluorescence plate reader with the appropriate filters (ex/em 485/535 nm) (Spectrophotometer Infinite M200, Tecan, Switzerland). Two biological replicates for each treatment (negative control and Rhinosectan^®^) were used for fluorescence evaluation.

### Statistical analysis

A descriptive analysis of quantitative data was performed. Mean and standard deviation values were calculated from Rhinosectan^**®**^ and saline-treated cell monolayers. Student’s T test was used to compare results between the two conditions. p values lower than 0.05 were considered significant.

## Results

### Rhinosectan^®^ improves the cilia beating frequency

**Fig. 2:**
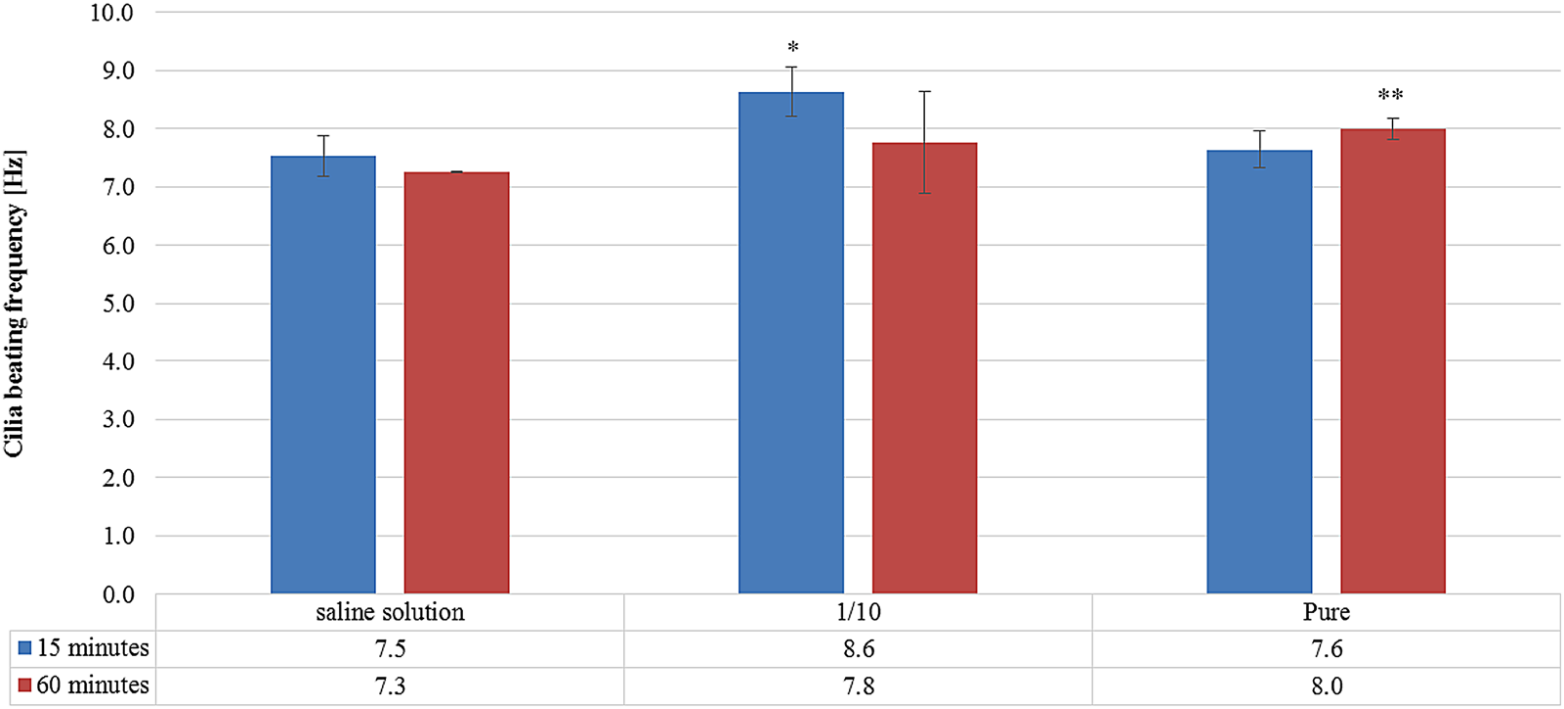
Cilia beating frequency assay. Comparative cilia beating frequency analysis of the MucilAir™ epithelium tissue upon exposure with undiluted or tenfold diluted Rhinosectan^**®**^ and a saline solution during 15 and 60 min. Experiments were performed in triplicate. Asterisks show p values: **p < 0.01, *p < 0.05 in comparison with saline solution (Student T test)

To test the effect of Rhinosectan^**®**^ on the stimulation of ciliary beating of ciliated respiratory cells, the MucilAir™ epithelium was exposed to Rhinosectan^**®**^ (diluted or undiluted). After 15 and 60 min of treatment, the cilia beating frequency of the epithelium tissue was measured (Fig. 2). The expected activity of the untreated monolayer was 7.2 ± 0.3 Hz (as reported in the certificate of analysis). In comparison with this value, no significant differences in the physiological activity of the cells were observed when treated with a saline solution as control (7.5 ± 0.4 and 7.3 ± 0.0 Hz at 15 and 60 min, respectively).

Upon application of Rhinosectan^**®**^ (diluted and undiluted), slight increases in cilia beating frequency were observed at both time points (15 and 60 min) in comparison with saline solution, indicating normal physiological activity of the nasal epithelium in the presence of Rhinosectan^**®**^ (Fig. 2). With undiluted Rhinosectan^**®**^, mean cilia beating frequency was 7.6 ± 0.13 Hz (p = 0.7) at 15 min and 8.0 ± 0.2 Hz at 60 min (p < 0.01), while with diluted product mean cilia beating frequency was 8.6 ± 0.4 Hz after 15 min (p < 0.05) and 8.0 ± 0.2 after 60 min (p < 0.01, in comparison with saline solution) (Fig. 2).

### Exposure of Rhinosectan^®^ on Mucilair™ reduces mucin secretion

**Fig. 3:**
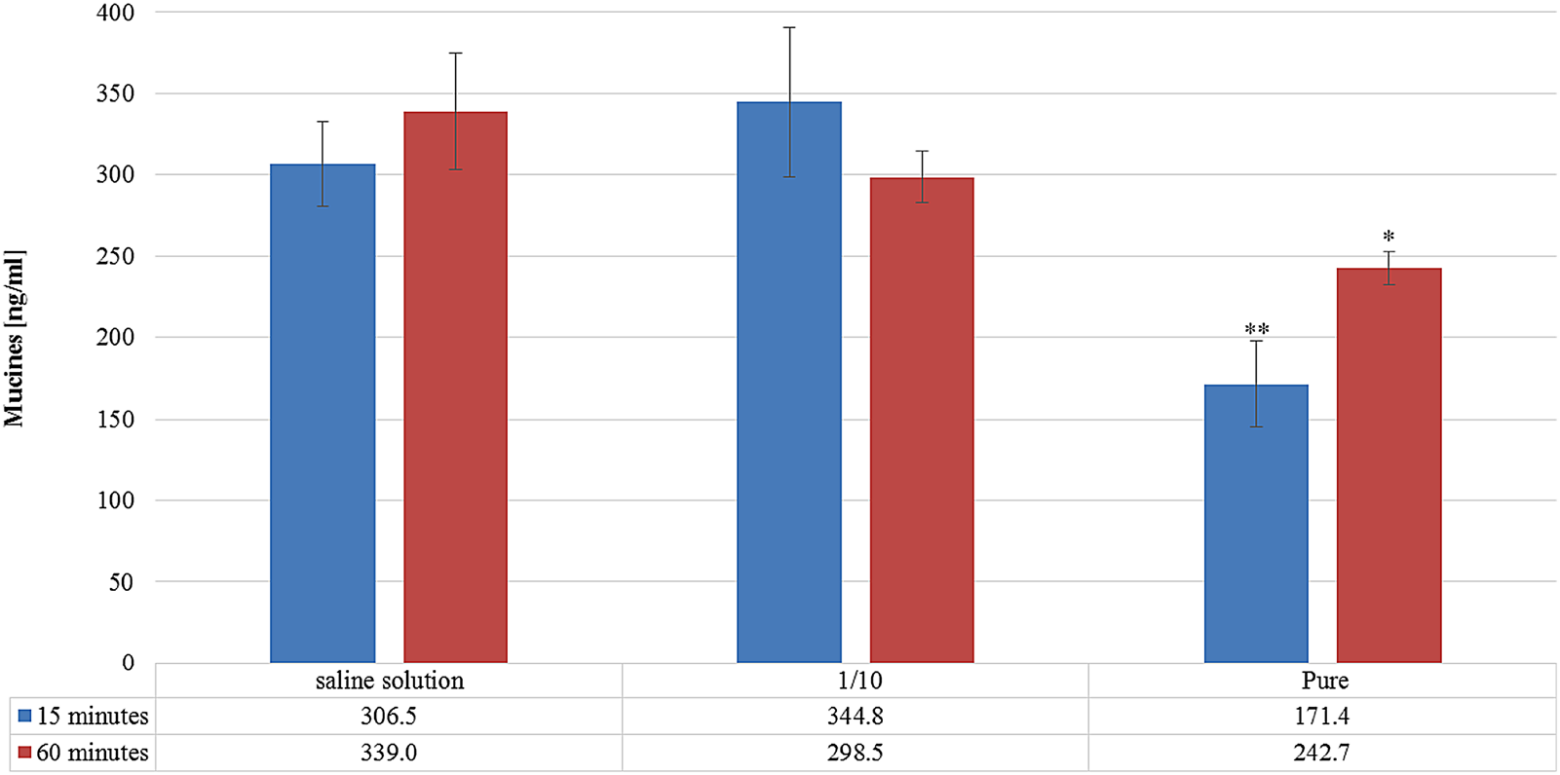
Mucin secretion. Effect of diluted and undiluted Rhinosectan^**®**^ on mucin secretion after exposure of the MucilAir™ epithelium to Rhinosectan^**®**^ during 15 and 60 min. The average of three independent experiments and standard deviation bar is shown. Asterisks show p values: **p < 0.01, *p < 0.05 in comparison with saline solution (Student T test)

The effect of Rhinosectan^**®**^ on mucin secretion by MucilAir™ tissue was measured with ELLA. After application of 30 µl of Rhinosectan^**®**^ or the negative control saline solution, the cell monolayers were incubated for 15 or 60 min and the secreted mucin was collected and quantified. As shown in Fig. 3, no statistically significant differences were observed between saline solution and tenfold diluted Rhinosectan^**®**^ on the production of mucin at both time points of 15 and 60 min (p = 0.27 and p = 0.24, respectively). Of note, an important decrease (almost 50%) in mucin concentration was observed with undiluted Rhinosectan^**®**^ after 15 min of exposure, in comparison with saline solution (171.4 ng/ml vs. 306.5 ng/ml; p < 0.01), and also after 60 min of exposure, with an approximated decrease of 25% (242.7 ng/ml vs. 339 ng/ml; p < 0.05).

### Exposure of Rhinosectan^®^ during 60 min increases mucociliary clearance

The effect of Rhinosectan^®^ on mucociliary clearance was evaluated on MucilAir™ epithelium and measurements of the movement of plugs of mucus on the apical surface were assayed.

**Fig. 4:**
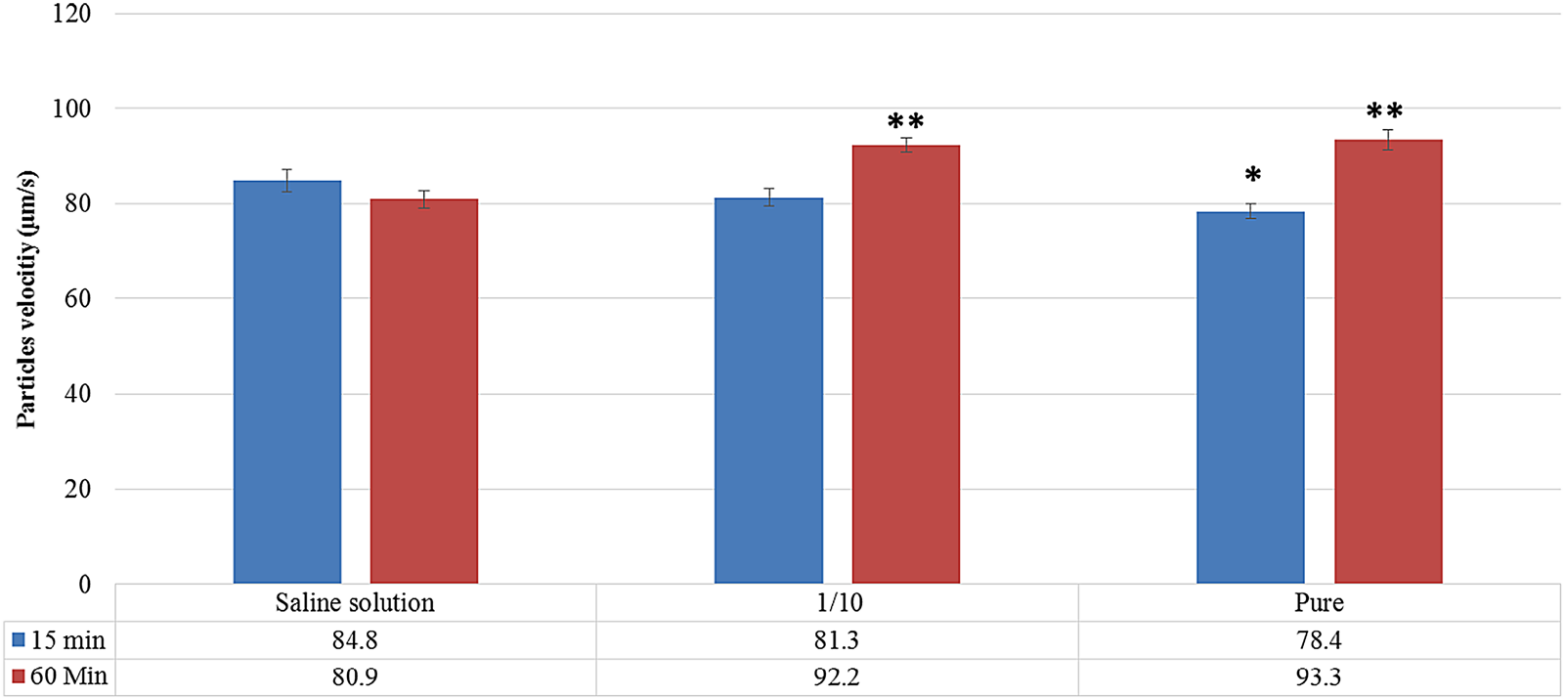
Mucociliary clearance. Mucociliary clearance analysis (MCC) of the epithelium tissue upon exposure during 15 and 60 min with diluted or undiluted Rhinosectan^**®**^ and a saline solution. The average of three independent experiments and standard deviation bar is shown. Asterisks show p values: **p < 0.01, *p < 0.05 in comparison with saline solution (Student T test)

At 15 min post-exposure, similar mucociliary clearance was observed with the 1/10 diluted sample of Rhinosectan^®^ (81.3 ± 1.9 μm/s) and saline solution (84.8 ± 2.4 μm/s), with a slight decrease with undiluted Rhinosectan^®^ (78.4 ± 1.6 μm/s; p < 0.05 vs. saline solution) (Fig. 4).

In contrast, at 60 min post-exposure, a marked increase in mucociliary clearance was observed with Rhinosectan^®^ (93.3 ± 2.1 μm/s with undiluted Rhinosectan^**®**^ and 92.2 ± 1.4 μm/s with diluted Rhinosectan^**®**^) (p < 0.01 compared to saline solution, 80.9 ± 1.8 μm/s) (Fig. 4).

### Rhinosectan^®^ enhances the phagocytic activity of nasal epithelial cells

**Fig. 5:**
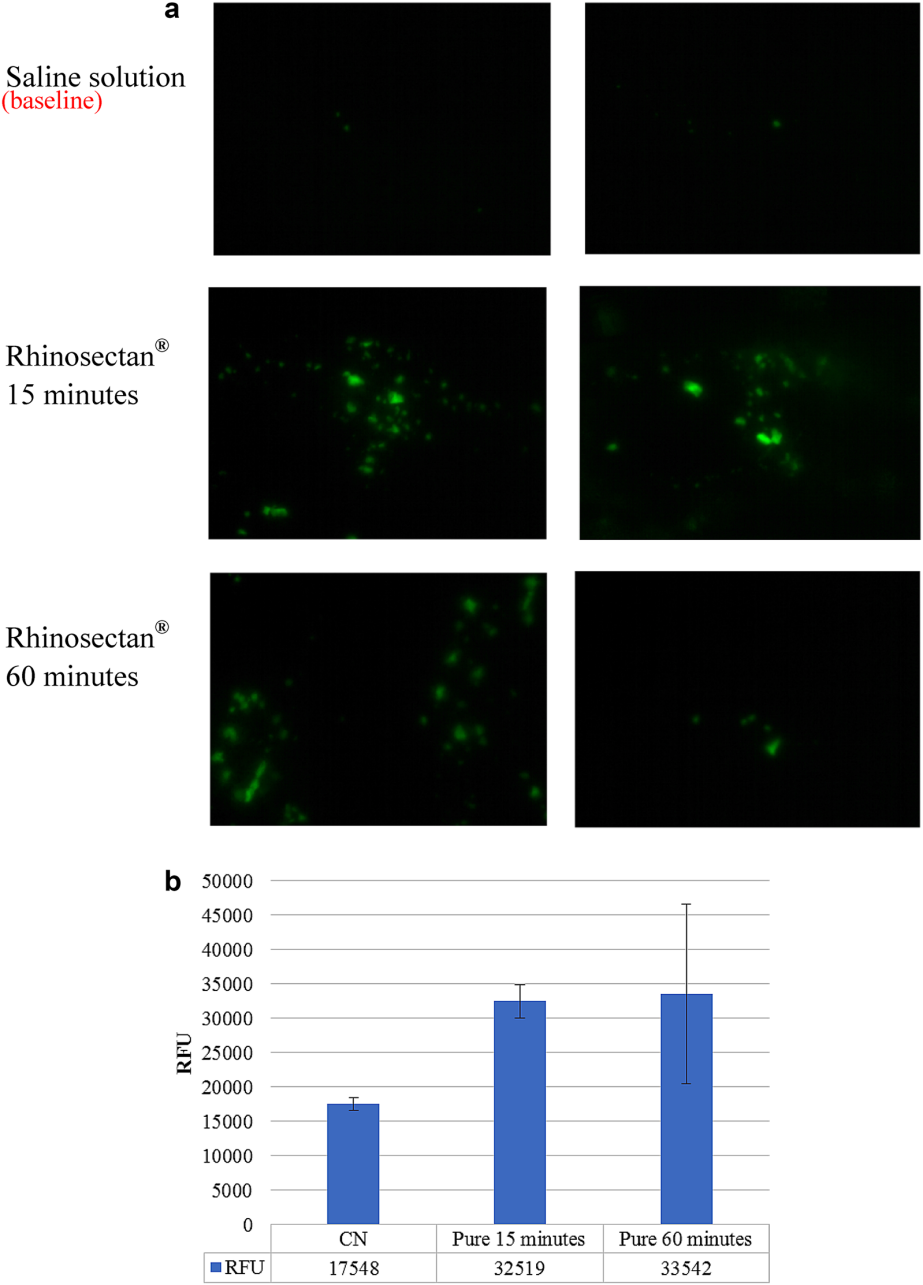
Phagocytic activity. Phagocytic activity of MucilAir™ cell monolayer treated with undiluted Rhinosectan^**®**^ after 15 and 60 min. 0.9% NaCl saline solution was used as a negative control. Latex Beads-Rabbit IgG-FITC solution was added to the cell monolayer at a 1:10 dilution simultaneously with 30 μl of Rhinosectan^®^ or saline solution and incubated at 37 °C for 15 or 60 min. Cells were washed with assay buffer and **a** visualized at ×10 magnification with the inverted fluorescence microscope or **b** the fluorescence intensity of the cells was measured in a fluorescence plate reader with the appropriate filter sets (ex/em 485/535 nm). **a** Duplicate tissues have been used for fluorescence evaluation but only a selection of images is reported. The first two images correspond to saline solution at baseline, the second ones to Rhinosectan at 15 min post-treatment and third ones to Rhinosectan at 60 min post-treatment. **b** The average of two independent experiments and standard deviation bars are shown. Asterisk show p-values lower than 0.05 (Student T test)

Phagocytic activity of MucilAir™ cell monolayer treated with Rhinosectan^**®**^ after 15 and 60 min was qualitatively and quantitatively evaluated (Fig. 5).

Compared to the cultures treated with saline solution, Rhinosectan^**®**^ treated cells showed a significant increase in the phagocytosis of fluorescent beads (green cells) after 15 min of exposure (Fig. 5a). Quantification of the fluorescence intensity of the cultures showed a 1.86-fold increase of the phagocytic activity of the Rhinosectan^**®**^ treated cells in comparison with saline solution at 15 min post-exposure (p < 0.05) (Fig. 5b). At 60 min post-treatment, a 1.89-fold increase in the phagocytic activity with Rhinosectan^**®**^ was detected, although with high variability (Fig. 5b).

## Discussion

Nowadays, there is increasing interest in assessing the role that the mucosal barrier plays in human health as it is the primary defense against pathogens, providing a critical transition between the external environment and the internal human body [1, 19].

In the case of respiratory diseases such as rhinitis, understanding the regulation of airway epithelial barrier function is opening potential new therapeutic targets [1, 5, 6]. Allergic rhinitis is a very common disorder that is frequently ignored, underdiagnosed, misdiagnosed, and mistreated. Undertreated allergic rhinitis underlies many complications, exacerbates asthma and is a major factor in asthma development, affecting quality of life and productivity at work or school [9, 20].

Management of rhinitis involves diagnosis, followed by avoidance of relevant allergens, with additional pharmacotherapy needed for most sufferers. According to severity, saline sprays non-sedating antihistamines (oral or topical, with minimally bioavailable intranasal corticosteroids for moderate/severe disease), possibly plus additional antihistamines or antileukotrienes can be used. The concept of rhinitis control is emerging, but there is no universally accepted definition. Where pharmacotherapy fails, allergen-specific immunotherapy, which is uniquely able to alter long-term disease outcomes, should be considered [20].

In this context, there is an increasing interest in the use of non-pharmacological measures to avoid allergens and other triggering factors as an integral part of the management strategy (as recommended by the ARIA guidelines) [5, 7, 8] and also to avoid the secondary effects of chronic pharmacological measures; thereby reducing the use of intranasal glucocorticosteroids and antihistamines [5, 8, 9, 21]. Although they have been established as the first-line therapies in allergic rhinitis, there is also an ongoing debate regarding their potential side effects on the nasal structure and ciliary function [16].

In this study, we have assessed the effects of the non-pharmacological spray Rhinosectan^®^, which contains xyloglucan, on a MucilAir™ nasal epithelial cell culture model. We have demonstrated that Rhinosectan^®^ does not interfere with normal nasal functioning, for example, in cilia beating frequency, while providing favorable effects such as reducing mucin secretion, improving mucociliary clearance and facilitating the phagocytic activity of nasal epithelial cells.

We did not detect alterations in the cilia beating frequency after exposure to Rhinosectan^®^. Cilia beat frequency is one of the basic functional parameters quantifying mucociliary activity and an important defense mechanism of the respiratory tract. For this reason, investigation of the influence of drugs on this parameter is of great importance in evaluating the tolerability and safety of nasally administered agents and preservatives, present in intranasal preparations employed in the management of nasal diseases [16].

Since impairment of ciliary movement is frequently associated with respiratory diseases such as rhinitis, sinusitis, bronchitis and otitis media [22], a prerequisite for products used in the management of rhinitis, sinusitis and related allergic or chronic conditions is that agents in the formulation do not interfere with normal nasal functioning [16].

These results are in contrast with studies showing interference of the ciliary movement in presence of nasal pharmacological agents: undiluted aqueous budesonide has shown to cause rapid but reversible ciliostasis [16], while undiluted or 50% dilution of fluticasone propionate and azelastine induced an irreversible ciliostatic effect [16, 23]. In our study, xyloglucan and the rest of components in the formulation of Rhinosectan^®^ and saline solution (the mainstay of the therapeutic armamentarium for patients with upper airway infections) did not alter the ciliary movement in the MucilAir™Nasal model at the time points evaluated (15 and 60 min, post-exposure), although further in vitro studies should be performed to evaluate the effect of Rhinosectan^®^ during the dosing interval (6–8 h).

Our results are also in line with a previous study performed with Rhinosectan^®^ in MucilAir™Nasal cells, in which Rhinosectan^®^ exhibited protective barrier properties, maintaining the location of tight junctions proteins and preserving the paracellular flux [5].

We also observed a significant increase in mucociliary clearance at 60 min post-exposure in comparison with saline solution. This increase was not observed at 15 min post-exposure, maybe due to the time required by xyloglucan to initiate the bioadhesion process on the mucous membranes (with mucin-like structure), owing to its high swelling capacity, which gives it its bio-protective film properties [1].

Mucociliary clearance removes inhaled and deposited particles, such as dust, allergens, bacteria and air pollutants, being essentially dependent upon the unidirectional beating of epithelial cilia and the rheological (visco-elastic) properties of mucus [16, 24]. Impairment of mucociliary clearance results in the accumulation of respiratory secretions and reduced defenses, leading to infections and inflammation [25]. Mucociliary clearance thus functions as a biomarker of nasal mucosal function [26].

In the case of rhinosinusitis, mucociliary clearance has been shown to be impaired due to both decreased ciliary beat frequency and altered mucus rheology [24]. Therefore, according to the results of this study, in the absence of ciliostasis, the improvement in mucociliary clearance together with the observed decrease in mucin concentration, suggests that Rhinosectan^®^ has optimal properties for use in the management of these disorders. In the case, for example, of hypertonic saline solutions, improvement of mucociliary clearance has also been observed, although a certain degree of ciliostasis may occur as a result of hypertonicity [24].

We also observed a significant increase in the phagocytic capacity after Rhinosectan^**®**^ exposure, at both 15 and 60 min post-treatment, suggesting a positive effect of the spray enhancing the ability of the epithelial cells to display their phagocytic function by efficiently trapping and removing inhaled particles and allergens.

Another important result of this study is the reduction in mucin secretion with the application of Rhinosectan^®^ spray, particularly when undiluted. Excessive mucus or impaired clearance contributes to the pathogenesis of all the common airway diseases [27–30]. When mucin production is increased, so that mucins accumulate intracellularly, and secretion of a large number of granules is then triggered (mucus hypersecretion), airway luminal occlusion can occur [31–33]. Additionally, persistent accumulation can lead to infection and inflammation by providing an environment for microbial growth.

Thus, the effect of Rhinosectan^®^ on mucin secretion might indicate a positive role in the regulation of the secretion, which is crucial for essential nasal functions although further studies are needed on this issue and taking into account that oligomeric mucins alone do not constitute mucus, and other mucin and non-mucin components must be important contributors to mucus organization and hence airways defense [27]. This is also in agreement with the results of the recent randomized, double-blind clinical trial performed in patients with rhinosinusitis, in which the application of Rhinosectan^®^ significantly improved main symptoms and reduced the severity of rhinosinusitis in comparison with a physiological saline nasal spray [10].

Therefore, these results suggest that application of Rhinosectan^®^ contributes to the optimal rheological (visco-elastic) properties of mucus by enhancing mucociliary clearance, taking into account that the balance between mucin production and clearance depends on optimal mucin quantities, the hydration state and periciliary fluid depth [34]. It is also important to note that stimulation of mucin secretion occurs with exposure to irritants and pro-inflammatory compounds and may lead to mucus obstruction of small airways and increased nasal resistance. It has been observed that products commonly used to relieve symptoms of chest congestion (containing products such as camphor, menthol and eucalyptus oil) can adversely affect mucociliary function, increasing mucin secretion and decreasing mucus clearance [35].

Although more aspects deserve further research, as further in vivo studies and clinical trials in patients with rhinitis during longer periods of time (for example during pollen seasons), the results of this study in the in vitro model of MucilAir™Nasal suggest that the application of Rhinosectan^®^ does not impair ciliary movement, enhances mucociliary clearance and facilitates phagocytosis, while reducing mucin secretion, which are optimal properties for the management of rhinitis and associated conditions.

## Abbreviations

3D: 3-dimensional
ALI: air–liquid interface
ARIA: allergic rhinitis and its impact on asthma
CBF: cilia beating frequency
ELLA: Enzyme-Linked Lectin Assay
FITC: fluorescein iso tiocyanate
HRP: horseradish peroxidase
IgG: immunoglobulin G
MCC: mucociliary clearance
PCI: Peripheral Component Interconnect
RFU: relative fluorescence units
TNSS: total nasal symptom score
